# Association of Benzodiazepine Treatment for Sleep Disorders With Drug Overdose Risk Among Young People

**DOI:** 10.1001/jamanetworkopen.2022.43215

**Published:** 2022-11-22

**Authors:** Greta A. Bushnell, Tobias Gerhard, Katherine Keyes, Deborah Hasin, Magdalena Cerdá, Mark Olfson

**Affiliations:** 1 Center for Pharmacoepidemiology and Treatment Sciences, Rutgers Institute for Health, Health Care Policy and Aging Research, New Brunswick, New Jersey; 2 Department of Biostatistics and Epidemiology, Rutgers University School of Public Health, Piscataway, New Jersey; 3 Department of Pharmacy Practice and Administration, Ernest Mario School of Pharmacy, Rutgers University, Piscataway, New Jersey; 4 Department of Epidemiology, Columbia University Mailman School of Public Health, New York, New York; 5 Department of Psychiatry, Vagelos College of Physicians and Surgeons, Columbia University Irving Medical Center, New York, New York; 6 Center for Opioid Epidemiology and Policy, Department of Population Health, New York University School of Medicine, New York, New York

## Abstract

**Importance:**

Benzodiazepines are prescribed for the treatment of adolescent sleep disorders; however, benzodiazepine overdoses occur, often in combination with opioids.

**Objective:**

To evaluate whether benzodiazepine treatment for sleep disorders, compared with alternative pharmacologic treatments (trazodone, hydroxyzine, zolpidem, zaleplon, and eszopiclone), is associated with increased risk of drug overdose for young people.

**Design, Setting, and Participants:**

This cohort study included privately insured people 10 to 29 years of age identified from a US commercial claims database (MarketScan), from January 1, 2009, to December 31, 2018. Young people with a sleep disorder diagnosis initiating benzodiazepine (n = 23 084) or comparator pharmacologic treatments (n = 66 706) were included in the study. Statistical analysis was performed from November 1, 2021, to May 16, 2022.

**Exposures:**

New use of benzodiazepine treatment or comparator pharmacologic treatments (defined as ≥1 year without a prescription for benzodiazepine or comparator medications).

**Main Outcomes and Measures:**

Incident diagnosed drug overdoses were identified from inpatient and emergency department records within 6 months of treatment initiation. The propensity score–adjusted cumulative incidence of overdose and hazard ratios (HRs) were estimated with intention-to-treat (analyzed based on initial treatment) and as-treated analyses (added censoring at treatment discontinuation). Results were stratified by prior prescription opioid fill.

**Results:**

The cohort included 23 084 young people initiating benzodiazepine treatment (14 444 female participants [62.6%]; mean [SD] age, 23 [4.1] years) and 66 706 initiating a comparator treatment (38 446 female participants [57.6%]; mean [SD] age, 22 [4.4] years). Six months after treatment initiation, 9.7% (95% CI, 9.3%-10.1%) of benzodiazepine users and 12.3% (95% CI, 12.1%-12.6%) of the comparator group were still receiving treatment. The crude incidence of drug overdose at 6 months was 0.9% for benzodiazepine initiators and 0.8% for comparator treatment initiators. In adjusted analyses, an increased risk of drug overdose was associated with benzodiazepines vs comparator treatments (intention-to-treat analysis: HR, 1.25 [95% CI, 1.03-1.51]; as-treated analysis: HR, 1.44 [95% CI, 1.14-1.80]). This association was stronger among young people with a recent prescription opioid fill vs those without a recent prescription opioid fill (as-treated analysis: adjusted HR, 2.01 [95% CI, 1.24-3.25] vs adjusted HR, 1.31 [95% CI, 1.00-1.70]).

**Conclusions and Relevance:**

The findings of this study suggest that benzodiazepines, compared with alternative pharmacologic treatments for common sleep disorders, were associated with an increased risk of drug overdose among young people during the following 6-month period, especially among those with a recent opioid prescription. Drug overdose is an important safety consideration when treating young people with benzodiazepines.

## Key Points

**Question:**

Is benzodiazepine treatment for sleep, compared with alternative pharmacologic treatments (trazodone, hydroxyzine, and sedative-hypnotic Z-drugs), associated with increased risk of overdose among young people?

**Findings:**

In this cohort study of 23 084 young people initiating benzodiazepine treatment and 66 706 initiating a comparator treatment, the risk of drug overdose in the 6 months after treatment start was elevated for young people starting benzodiazepine treatment compared with alternative treatments for sleep disorders. This risk was further heightened for young people with a recent prescription opioid fill.

**Meaning:**

The findings of this study suggest that the elevated risk of drug overdose with benzodiazepine treatment compared with alternative pharmacologic treatments for sleep disorders is an important safety consideration when treating young people.

## Teaser

This cohort study evaluates whether benzodiazepine treatment for sleep disorders, compared with alternative pharmacologic treatments (trazodone, hydroxyzine, zolpidem, zaleplon, and eszopiclone), is associated with increased risk of drug overdose for young people.

## Introduction

Between 1991 and 2019, inadequate sleep among adolescents in the United States increased from 44% to 66%.^1^ Insomnia is associated with negative health consequences.^2,3,4^ Nonpharmacologic and pharmacologic treatments for insomnia are available, with cognitive behavioral therapy frequently recommended as first-line treatment.^5,6^ There are also several prescription pharmacologic treatments, although many have limited or low-quality evidence of effectiveness or safety, particularly for longer-term treatment.^5,7^ For children (<18 years), there are even fewer data on safety and tolerability of prescription insomnia treatments.^8^

Benzodiazepines are 1 class of medications prescribed for the treatment of sleep disorders,^9,10,11,12,13^ with selected benzodiazepines approved by the US Food and Drug Administration for insomnia in adults (≥18 years). Although benzodiazepines are commonly prescribed, including to young people,^11,14,15,16^ they are recommended less frequently for insomnia among children than among adults given the lack of efficacy and safety data for younger age groups.^8,17^ When benzodiazepines are prescribed for any age group, short-term treatment (≤4 weeks) is recommended.^6^

Serious risks of benzodiazepines include nonmedical use, benzodiazepine use disorders, and overdose.^15,18,19,20^ Because the risk of overdose increases when benzodiazepines are used in combination with opioids and other central nervous system depressants,^21,22,23^ benzodiazepine-attributed morbidity and mortality have increased since the onset of the prescription opioid epidemic.^15^ In 2020, benzodiazepines were associated with 12 290 overdose deaths, an increase from 6872 in 2011 and 1135 in 1999.^24^ Persons with fatal and nonfatal benzodiazepine-related overdoses frequently have had prior prescriptions for benzodiazepines.^25,26^ In a comparative safety study of adults, benzodiazepine treatment was associated with elevated risk of all-cause mortality compared with selective serotonin reuptake inhibitor treatment.^27^ Risks of drug overdose after benzodiazepine treatment for insomnia were unclear, with no comparative evidence among young people.

Adolescence and young adulthood are critical periods to consider drug overdose risk after prescription benzodiazepine treatment. Initial exposure to alcohol and other drugs often occurs in adolescence,^28,29^ nonmedical prescription drug use increases during this developmental period,^30,31^ and prescription benzodiazepine misuse increases, with 1.5% of youths (12-17 years) and 3.8% of young adults (18-25 years) reporting past-year prescription benzodiazepine misuse.^32^ Given the availability of alternative pharmacologic treatments for sleep disorders,^7,33^ it is important to examine whether increased overdose risk is associated with benzodiazepine treatment compared with commonly prescribed alternative medications.

We therefore sought to evaluate whether benzodiazepine treatment compared with alternative prescription treatments for sleep disorders among young people was associated with increased drug overdose risk in the 6 months after treatment start, and whether this association varies by recent prescription opioid use. We hypothesized that, for young people, benzodiazepine treatment would be associated with an increased risk of drug overdose compared with alternative sleep medications.

## Methods

### Data Source and Study Population

The study cohort was identified from the 2009-2018 MarketScan US commercial claims database, which includes patient-level records on insurance enrollment details; dispensed prescription medications; and inpatient, outpatient, and emergency department (ED) health care encounters.^34^ The study population included privately insured young people aged 10 to 29 years. We identified young people with a sleep disorder diagnosis newly initiating benzodiazepine treatment or an alternative pharmacologic treatment. New use was defined with a 1-year washout period without a prior benzodiazepine prescription, an alternative pharmacologic treatment (sedative-hypnotic Z-drugs [ie, zolpidem, zaleplon, and eszopiclone], hydroxyzine, trazodone), or clonidine. Clonidine, which is often used for sleep disorders in younger adolescents, was included in the washout period but was not included as an alternative pharmacologic treatment given its infrequent use for insomnia among young adults. Continuous insurance enrollment with prescription drug coverage was required in the prior year. The Rutgers University institutional review board approved this study under expedited review. A waiver of consent was granted because the data were deidentified. Our report followed the Strengthening the Reporting of Observational Studies in Epidemiology (STROBE) reporting guideline for observational studies.

We required a diagnosis of insomnia (*International Classification of Diseases, Ninth Revision, Clinical Modification* [*ICD-9-CM*] codes 307.41, 307.42, 327.0x, and 780.52; *International Statistical Classification of Diseases and Related Health Problems, Tenth Revision, Clinical Modification* [*ICD-10-CM*] codes F51.0x and G47.0x) or unspecified sleep disorder (*ICD-9-CM* codes 307.40 and 780.50; *ICD-10-CM* codes F51.9x and G47.9x) (hereafter referred to as *sleep disorders*) 30 or less days prior to treatment initiation. Additional cohort exclusions included clinical contraindications (eg, sleep apnea), epilepsy diagnosis, combination treatments, trazodone initiation at higher doses, and initiating treatment with 1 or more medication classes (eFigure 1 in the Supplement).

### Benzodiazepines and Alternative Pharmacologic Treatments

Prescription benzodiazepine use was defined as a dispensed prescription for alprazolam, chlordiazepoxide, clobazam, clonazepam, clorazepate, diazepam, estazolam, flurazepam, lorazepam, midazolam, oxazepam, quazepam, temazepam, and triazolam, excluding combination products. We used an active comparator design. Our comparator was initiation of alternative prescription sleep disorder treatments, including multiple medication classes.^35^ With no single ideal comparator, the comparator group was selected based on treatment guidelines and prescribing practices.^7,13,17,36,37,38,39^ The comparator group included trazodone, hydroxyzine, and Z-drugs. We excluded young people initiating trazodone (>150 mg/d) given the likelihood it was for depression. For a sensitivity analysis, the comparator group was expanded to include new users of clonidine.

### Drug Overdose

In the 6 months after treatment initiation, we identified incident drug overdoses from inpatient or ED records. The primary outcome, any drug overdose, was defined with *ICD* codes for unintentional, intentional, and undetermined drug poisonings (eTable 1 in the Supplement); *ICD-10-CM* codes were restricted to codes for initial encounters. We also separately examined overdoses recorded to involve benzodiazepines (*ICD-9-CM* codes 969.4 and E853.2; *ICD-10-CM* codes T42.4X1A, T42.4X2A, and T42.4X4A). Both outcomes capture primarily nonfatal overdose events; fatal overdoses were included if patients were brought to an ED or hospital.

### Additional Patient Characteristics

Patient-level variables were included to describe the study population and as confounders. These included demographic characteristics (age and sex), comorbid psychiatric diagnoses, psychotropic medication prescriptions, other prescription medications, nonpsychiatric diagnoses including injury and poisoning codes, and health care use measures. We also included the clinician type associated with the sleep disorder diagnosis, contact with a mental health professional, and psychotherapy claims. Recent opioid prescription, a stratification variable, was defined as 1 or more opioid prescriptions dispensed 90 or less days before benzodiazepine or comparator treatment start.

### Statistical Analysis

Statistical analysis was performed from November 1, 2021, to May 16, 2022. We characterized the study population and compared benzodiazepine and comparator initiators. We estimated treatment length, with discontinuation defined as no prescription fill for 30 days after the last prescription supply ended. Discontinuation in the comparator group was defined as no subsequent prescription for any medication within the comparator group. We then estimated the cumulative incidence of 3- and 6-month overdose with the Kaplan-Meier estimator, and the hazard ratios (HRs) and 95% CIs using Cox proportional hazards regression models.

Propensity scores were estimated with logistic regression and applied using inverse probability of treatment weighting (IPTW) to estimate the adjusted cumulative incidence and HRs. The potential confounders and the factors associated with drug overdose were included in the propensity score model (eTable 2 in the Supplement). Inverse probability of treatment weights were applied to the Cox proportional hazards regression model, and no other covariates were added. In the primary analysis, minimal trimming was applied to areas with no propensity score overlap. Doubly robust 95% CIs were estimated for adjusted HRs.

As-treated and intention-to-treat (ITT) cumulative incidences and HRs are presented. In ITT analyses, youths were followed up until overdose, 6 months, insurance disenrollment, or end of data (December 31, 2018), whichever came first. In as-treated analyses, youths were followed up until treatment discontinuation (30-day grace period), overdose, 6 months, insurance disenrollment, or end of data, whichever came first. Individuals were analyzed based on initial treatment. Intention-to-treat estimates were included because overdose risk may continue after treatment ends (eg, if treatment was associated with subsequent substance misuse). As-treated analyses provide risk estimates during initial treatment and are useful for safety studies.

#### Secondary Analyses

We conducted 2 secondary analyses with propensity scores recreated within each stratum. We stratified by 1 or more filled opioid prescriptions in the 90 days before treatment start. We also stratified by age group: young adolescents (10-17 years) and young adults (18-29 years); post hoc analyses were stratified by ages 18 to 24 years and 25 to 29 years.

#### Sensitivity Analyses

We estimated results using stabilized IPTW (SIPTW) and applied additional trimming for the IPTW-adjusted analysis, at the first percentile of the propensity score in the exposed group and the 99th percentile of the unexposed group. We stratified by antidepressant use 90 or less days before benzodiazepine or comparator treatment start. We conducted analyses excluding individuals aged 10 to 11 years and including new users of clonidine in the comparator group. In a post hoc sensitivity analysis, we assessed the strength necessary for an unmeasured confounder to fully explain the observed association between treatment type and drug overdose if no association existed.^40^

## Results

### Study Cohort

The primary cohort included 23 084 patients initiating benzodiazepine treatment (14 444 female participants [62.6%]; mean [SD] age, 23 [4.1] years) and 66 706 initiating comparator treatments (38 446 female participants [57.6%]; mean [SD] age, 22 [4.4] years; trazodone, 27 815; hydroxyzine, 10 929; and Z-drugs, 27 962), with 90.3% (81 097 of 89 790) having an insomnia diagnosis and 9.7% (8693 of 89 790) an unspecified sleep disorder diagnosis (eTable 2 in the Supplement).

New users of benzodiazepines and new users of comparator drugs had many similar characteristics (Table 1). Benzodiazepine initiators compared with the comparator group were more likely to have had recent selective serotonin reuptake inhibitor use (8629 of 23 084 [37.4%] vs 18 547 of 66 706 [27.8%]) and unspecified anxiety diagnoses (6897 of 23 084 [29.9%] vs 10 193 of 66 706 [15.3%]) and were slightly less likely to have had prior substance use disorder diagnoses (alcohol use disorder, 462 of 23 084 [2.0%] vs 1620 of 66 706 [2.4%]; cannabis use disorder, 404 of 23 084 [1.8%] vs 1631 of 66 706 [2.4%]; opioid use disorder, 253 of 23 084 [1.1%] vs 943 of 66 706 [1.4%]) or suicidal ideation diagnoses (394 of 23 084 [1.7%] vs 1894 of 66 706 [2.8%]). These differences were not present once propensity score IPTW was applied for adjusted analyses.

**Table 1.** Young People Initiating Benzodiazepine or Comparator Treatment for Sleep Disorder: Crude and Propensity Score–Weighted Cohorts^a^

Characteristic	Study cohort, unweighted			Inverse probability of treatment weighting^b^		
	No. (%)		Standardized difference	Weighted, No (%)		Standardized difference
	Benzodiazepine initiators (n = 23 084)	Comparator group (n = 66 706)		Benzodiazepine initiators (n = 89 515)	Comparator group (n = 89 814)	
Male	8640 (37.4)	28 260 (42.4)	0.10	36 674 (41.0)	36 812 (41.0)	0.00
Age at treatment initiation, y^c^						
10-17	2001 (8.7)	9730 (14.6)	0.19	11 865 (13.3)	11 713 (13.0)	0.01
18-24	11 424 (49.5)	33 481 (50.2)	0.01	44 712 (49.9)	44 868 (50.0)	0.00
25-29	9659 (41.8)	23 495 (35.2)	0.14	32 938 (36.8)	33 233 (37.0)	0.00
**Comorbid psychiatric diagnoses, 1 y**						
Anxiety disorder, unspecified						
Recent diagnosis (≤30 d)	6897 (29.9)	10 193 (15.3)	0.36	17 116 (19.1)	17 328 (19.3)	0.00
Past diagnosis only (31-365 d)	1110 (4.8)	3778 (5.7)	0.04	5125 (5.7)	4885 (5.4)	0.01
Major depressive disorder						
Recent diagnosis (≤30 d)	4045 (17.5)	12 586 (18.9)	0.04	17 011 (19.0)	16 625 (18.5)	0.01
Past diagnosis only (31-365 d)	1350 (5.8)	4260 (6.4)	0.02	5745 (6.4)	5639 (6.3)	0.01
Generalized anxiety disorder						
Recent diagnosis (≤30 d)	2660 (11.5)	4913 (7.4)	0.14	7670 (8.6)	7734 (8.6)	0.00
Past diagnosis only (31-365 d)	551 (2.4)	1872 (2.8)	0.03	2578 (2.9)	2419 (2.7)	0.01
Panic disorder	1357 (5.9)	1361 (2.0)	0.20	2782 (3.1)	2828 (3.1)	0.00
Other depressive disorder	1311 (5.7)	3171 (4.8)	0.04	4733 (5.3)	4539 (5.1)	0.01
Bipolar disorder	749 (3.2)	1898 (2.8)	0.02	2845 (3.2)	2683 (3.0)	0.01
Acute stress	635 (2.8)	923 (1.4)	0.10	1613 (1.8)	1584 (1.8)	0.00
Suicidal ideation diagnosis	394 (1.7)	1894 (2.8)	0.08	2340 (2.6)	2223 (2.5)	0.01
Self-harm diagnosis	71 (0.3)	478 (0.7)	0.06	472 (0.5)	499 (0.6)	0.00
Alcohol use disorder	462 (2.0)	1620 (2.4)	0.03	2121 (2.4)	2079 (2.3)	0.00
Cannabis use disorder	404 (1.8)	1631 (2.4)	0.05	2182 (2.4)	2040 (2.3)	0.01
Opioid use disorder	253 (1.1)	943 (1.4)	0.03	1263 (1.4)	1198 (1.3)	0.01
**Medications, 1 y**						
SSRI						
Recent prescription (≤90 d)	8629 (37.4)	18 547 (27.8)	0.21	27 352 (30.6)	27 444 (30.6)	0.00
Prior prescription only (91-365 d)	927 (4.0)	2856 (4.3)	0.01	3952 (4.4)	3789 (4.2)	0.01
Opioid (prescription)						
Recent prescription (≤90 d)	3504 (15.2)	8867 (13.3)	0.05	12 817 (14.3)	12 469 (13.9)	0.01
Prior prescription only (91-365 d)	4653 (20.2)	13 038 (19.5)	0.02	17 603 (19.7)	17 689 (19.7)	0.00
Skeletal muscle relaxant						
Recent prescription (≤90 d)	1054 (4.6)	2848 (4.3)	0.01	3944 (4.4)	3919 (4.4)	0.00
Prior prescription only (91-365 d)	1271 (5.5)	3766 (5.6)	0.01	5183 (5.8)	5050 (5.6)	0.01
SNRI	1183 (5.1)	3095 (4.6)	0.02	4581 (5.1)	4346 (4.8)	0.01
Non-SSRI or SNRI antidepressant	2450 (10.6)	6494 (9.7)	0.03	9225 (10.3)	9034 (10.1)	0.01
Stimulant	2198 (9.5)	7131 (10.7)	0.04	9366 (10.5)	9353 (10.4)	0.00
Antipsychotic	1164 (5.0)	2469 (3.7)	0.07	3970 (4.4)	3744 (4.2)	0.01
NSAID	4976 (21.6)	14 195 (21.3)	0.01	19 204 (21.5)	19 213 (21.4)	0.00
Antihistamine	1970 (8.5)	5558 (8.3)	0.01	7796 (8.7)	7562 (8.4)	0.01
**Health care use**						
Inpatient psychiatric admission, 1 y	677 (2.9)	2953 (4.4)	0.08	3766 (4.2)	3565 (4.0)	0.01
ED visit, recent (≤3 mo)	3392 (14.7)	8499 (12.7)	0.06	12 288 (13.7)	11 939 (13.3)	0.01
Psychotherapy claim, recent (≤30 d)	1941 (8.4)	5786 (8.7)	0.01	8337 (9.3)	7816 (8.7)	0.02
Psychiatry contact (≤90 d)	1535 (6.6)	4843 (7.3)	0.02	6742 (7.5)	6408 (7.1)	0.02
**Other diagnoses, 1 y**						
Musculoskeletal pain	5312 (23.0)	16 206 (24.3)	0.03	21 829 (24.4)	21 566 (24.0)	0.01
Fatigue, malaise	5228 (22.6)	12 528 (18.8)	0.10	17 871 (20.0)	17 855 (19.9)	0.00
Migraine, headache	4112 (17.8)	11 590 (17.4)	0.01	16 017 (17.9)	15 781 (17.6)	0.01
Low-back pain	2243 (9.7)	6486 (9.7)	0.00	8837 (9.9)	8741 (9.7)	0.01
Nonspecific chest pain	1890 (8.2)	4373 (6.6)	0.06	6484 (7.2)	6338 (7.1)	0.01
Syncope, dizziness	1543 (6.7)	3753 (5.6)	0.04	5404 (6.0)	5357 (6.0)	0.00
Pregnancy	1091 (4.7)	2326 (3.5)	0.06	3383 (3.8)	3430 (3.8)	0.00
IBS, Crohn disease	630 (2.7)	1608 (2.4)	0.02	2396 (2.7)	2279 (2.5)	0.01
Poisoning, adverse effect						
Recent poisoning (≤30 d)	203 (0.9)	744 (1.1)	0.02	916 (1.0)	925 (1.0)	0.00
Prior poisoning only (31-365 d)	182 (0.8)	564 (0.8)	0.01	792 (0.9)	761 (0.8)	0.00

Abbreviations: ED, emergency department; IBS, irritable bowel syndrome; NSAID, nonsteroidal anti-inflammatory drug; SNRI, serotonin-norepinephrine reuptake inhibitor; SSRI, selective serotonin reuptake inhibitor.

^a^ MarketScan 2009-2018 commercial claims database (full list of patient characteristics in eTable 2 in the Supplement).

^b^ A total of 153 new users in the comparator group were excluded from adjusted analysis from trimming.

^c^ Age of cohort by narrower groupings: 10 to 11 years (benzodiazepine [n = 107]; comparator [n = 526]), 12 to 17 years (benzodiazepine [n = 1894]; comparator [n = 9204]), 18 to 21 years (benzodiazepine [n = 5721]; comparator [n = 18 597]), 22 to 25 years (benzodiazepine [n = 7774]; comparator [n = 19 789]), and 26 to 29 years (benzodiazepine [n = 7588]; comparator [n = 18 590]).

### Treatment Duration

Most youths filled 1 prescription before discontinuing treatment (benzodiazepine group, 68.6% [15 843 of 23 084] with 1 fill; comparator group, 69.5% [46 353 of 66 706] with 1 fill). The median days’ supply for the initial prescription was 20 days (IQR, 10-30 days) for benzodiazepine new users and 30 days for the comparator group (IQR, 30-30 days). Three months after treatment start, 22.9% (95% CI, 22.4%-23.4%) of benzodiazepine users and 28.9% (95% CI, 28.6-29.1) of the comparator group were still receiving treatment; at 6 months, 9.7% (95% CI, 9.3%-10.1%) of benzodiazepine users and 12.3% (95% CI, 12.1%-12.6%) of the comparator group were still receiving treatment.

### Risk of Drug Overdose

During the 6-month follow-up, 684 drug overdose events occurred, including 190 among benzodiazepine initiators. At 6 months, the crude cumulative incidence of drug overdose was 0.9% for benzodiazepine initiators and 0.8% for the comparator group.

In the propensity score–weighted cohort, benzodiazepine and comparator initiators were balanced on measured covariates (standardized differences, ≤0.02) (Table 1; eTable 2 in the Supplement). The adjusted cumulative incidence of drug overdose at 6 months was 1.0% for young people starting benzodiazepine treatment for a sleep disorder and 0.8% for young people initiating comparator sleep medications (ITT analysis: HR, 1.25 [95% CI, 1.03-1.51]) (Table 2; eFigure 2 in the Supplement). In the as-treated analysis, the adjusted 6-month risk of drug overdose was 1.6% for the benzodiazepine group and 1.0% for the comparator group (HR, 1.44 [95% CI, 1.14-1.80]).

**Table 2.** Cumulative Incidence and Hazard Ratio of Drug Overdose Within 6 Months of Benzodiazepine or Comparator Treatment Initiation Among Young People With Sleep Disorders^a^

Analysis	Total No. (unweighted)	Drug overdose events^b^^,^^c^	Cumulative incidence, %			HR (95% CI)
			3 mo	6 mo	Difference at 6 mo	
**Intention-to-treat analysis**						
Crude						
Benzodiazepine treatment	23 084	190	0.6	0.9	0.1	1.11 (0.94-1.31)
Comparator treatment	66 706	494	0.5	0.8	1 [Reference]	1 [Reference]
Adjusted						
Benzodiazepine treatment	23 084	819.0	0.6	1.0	0.2	1.25 (1.03-1.51)
Comparator treatment	66 553	656.9	0.5	0.8	1 [Reference]	1 [Reference]
**As-treated analysis^d^**						
Crude						
Benzodiazepine treatment	23 084	128	0.7	1.4	0.5	1.31 (1.07-1.61)
Comparator treatment	66 706	313	0.5	1.0	1 [Reference]	1 [Reference]
Adjusted						
Benzodiazepine treatment	23 084	540.8	0.8	1.6	0.6	1.44 (1.14-1.80)
Comparator treatment	66 553	423.9	0.5	1.0	1 [Reference]	1 [Reference]

Abbreviation: HR, hazard ratio.

^a^ Young people aged 10 to 29 years with a diagnosis of insomnia or unspecific sleep disorder.

^b^ Weighted outcome counts within 6 months displayed for adjusted analyses.

^c^ Unweighted number of events in adjusted analysis within 3 months: intention-to-treat analysis (benzodiazepine, n = 122; comparator, n = 314), as-treated analysis (benzodiazepine, n = 107; comparator, n = 262), and within 6 months: intention-to-treat analysis (benzodiazepine, n = 190; comparator, n = 487), as-treated (benzodiazepine, n = 128; comparator, n = 308).

^d^ As-treated analysis added censoring at treatment discontinuation with a 30-day grace period.

### Risk of Overdose Involving a Benzodiazepine

During the 6-month follow-up, there were 116 overdoses associated with benzodiazepine, of which 70 were among benzodiazepine initiators. The crude cumulative incidence of overdose involving benzodiazepines was 0.3% among benzodiazepine initiators and 0.1% among the comparator group (adjusted ITT analysis: HR, 4.65 [95% CI, 3.13-6.91]) (eTable 3 in the Supplement).

### Prescription Opioid Stratification

In the 3 months before treatment initiation, 13.8% of the cohort had a prescription opioid dispensed. When stratifying by opioid prescription, the crude cumulative 6-month incidence of drug overdose was higher among benzodiazepine initiators with a recent opioid prescription (1.6%) (Figure) compared with benzodiazepine initiators without a recent opioid prescription (0.8%) and initiators of alternative sleep medications with (0.8%) and without (0.8%) a recent opioid prescription. The adjusted association between treatment type and drug overdose was heightened among youths with a recent prescription opioid dispensed (as-treated analysis: HR, 2.01 [95% CI, 1.24-3.25]) vs those without a recent opioid prescription (HR, 1.31 [95% CI, 1.00-1.70]) (Table 3).

**Figure.**
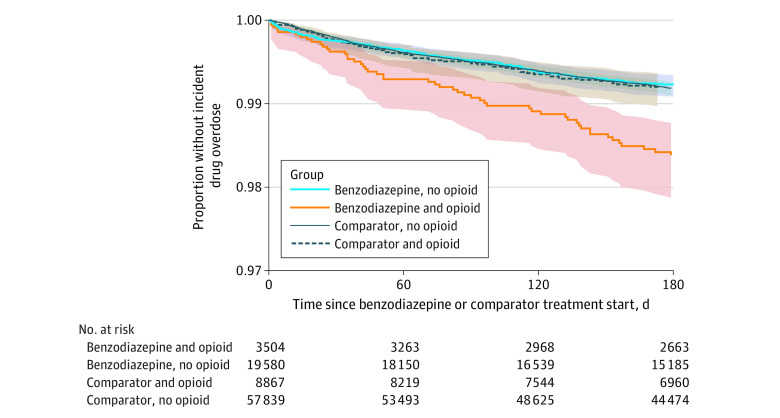
Crude Survival Function of Drug Overdose by Treatment Type and Prescription Opioid Status Intention-to-treat analysis; opioid prescription dispensing within the 90 days before benzodiazepine or comparator treatment start; 2009-2018 MarketScan commercial claims database. Shaded areas indicate 95% CIs.

**Table 3.** Secondary and Sensitivity Analyses Evaluating the Comparative Risk of Drug Overdose for Benzodiazepine Initiators vs Comparator Treatment Initiators: Adjusted Results

Secondary or sensitivity analysis	Total No. (unweighted)	As-treated analysis		Intention-to-treat analysis	
		Incidence at 6 mo, %	HR (95% CI)	Incidence at 6 mo, %	HR (95% CI)
Opioid prescription					
Recent opioid prescription					
Benzodiazepine treatment	3501	2.7	2.01 (1.24-3.25)	1.7	2.07 (1.37-3.12)
Comparator treatment	8863	0.9	1 [Reference]	0.8	1 [Reference]
No recent opioid prescription					
Benzodiazepine treatment	19 580	1.3	1.31 (1.00-1.70)	0.9	1.10 (0.88-1.36)
Comparator treatment	57 687	1.0	1 [Reference]	0.8	1 [Reference]
Age at treatment initiation^a^					
18-29 y					
Benzodiazepine treatment	21 083	1.4	1.63 (1.25-2.12)	0.9	1.48 (1.19-1.85)
Comparator treatment	56 962	0.7	1 [Reference]	0.6	1 [Reference]
**Sensitivity analyses**					
IPTW, extended trimming (1st percentile, 99th percentile)					
Benzodiazepine treatment	21 750	1.6	1.59 (1.26-2.01)	1.0	1.32 (1.09-1.59)
Comparator treatment	63 040	0.9	1 [Reference]	0.8	1 [Reference]
SIPTW					
Benzodiazepine treatment	23 084	1.6	1.41 (1.13-1.78)	1.0	1.24 (1.02-1.50)
Comparator treatment	66 706	1.0	1 [Reference]	0.8	1 [Reference]
Age 12-29 y					
Benzodiazepine treatment	22 977	1.6	1.45 (1.15-1.82)	1.0	1.26 (1.04-1.53)
Comparator treatment	66 058	1.0	1 [Reference]	0.8	1 [Reference]
Expanded comparison group					
Benzodiazepine treatment	23 076	1.6	1.50 (1.19-1.88)	1.0	1.29 (1.06-1.58)
Comparator treatment (with clonidine)	70 756	1.0	1 [Reference]	0.8	1 [Reference]
Antidepressant prescription					
Recent antidepressant prescription					
Benzodiazepine treatment	10 540	2.3	1.42 (1.05-1.92)	1.6	1.23 (0.97-1.57)
Comparator treatment	23 306	1.5	1 [Reference]	1.3	1 [Reference]
No recent antidepressant prescription					
Benzodiazepine treatment	12 543	1.1	1.64 (1.14-2.36)	0.7	1.32 (0.97-1.81)
Comparator treatment	43 182	0.6	1 [Reference]	0.5	1 [Reference]

Abbreviations: HR, hazard ratio; IPTW, inverse probability of treatment weighting; SIPTW, stabilized inverse probability of treatment weighting.

^a^ For young adolescents aged 10 to 17 years (n = 11 731), HRs are not estimated given nonproportional hazards in as-treated and intention-to-treat analyses. Cumulative incidence in adjusted intention-to-treat analysis: 90 days (benzodiazepine initiators, 1.2%; comparator treatment, 1.3%), 180 days (benzodiazepine initiators, 1.8%; comparator treatment, 2.1%). Cumulative incidence in adjusted as-treated analysis: 90 days (benzodiazepine initiators, 1.9%; comparator treatment, 1.4%), 180 days (benzodiazepine initiators, 3.3%; comparator treatment, 2.4%); benzodiazepine initiators had higher incidence of drug overdose beginning around 80 days after treatment start.

### Age Stratification

For young adults aged 18 to 29 years, the adjusted 6-month cumulative incidence of drug overdose (ITT analysis) was 0.9% for benzodiazepine initiators and 0.6% for comparator treatment initiators. In the adjusted analysis for young adults, benzodiazepine treatment was associated with an increased risk of drug overdose compared with comparator treatment (as-treated analysis: HR, 1.63 [95% CI, 1.25-2.12]) (Table 3). In post hoc stratification by ages 18 to 24 years and 25 to 29 years, the adjusted HRs were similar (as-treated analysis: HR, 1.62 [95% CI, 1.20-2.18] and 1.52 [95% CI, 0.87-2.66], respectively). However, in adjusted ITT analysis, the 6-month cumulative incidence of drug overdose was higher among persons aged 18 to 24 years compared with those aged 25 to 29 years (18-24 years: benzodiazepine, 1.2%; comparator, 0.8%; and 25-29 years: benzodiazepine, 0.5%; comparator, 0.3%).

When restricted to young adolescents aged 10 to 17 years, Kaplan-Meier analyses revealed intersecting survival functions; HRs were not estimated. Compared with young adults aged 18 to 29 years, cumulative incidence estimates of drug overdose were higher among young adolescents (adjusted ITT analysis: benzodiazepine initiators, 1.8%; comparator, 2.1%; Table 3).

### Sensitivity Analyses

Results were consistent implementing SIPTW, under IPTW with increased trimming of the propensity score distribution tails, excluding individuals aged 10 to 11 years, and expanding the comparator group (n = 4252) to include clonidine initiators (Table 3). The risk of a drug overdose remained elevated for benzodiazepine users vs comparators among youths with or without a recent antidepressant prescription. The post hoc sensitivity analysis depicts scenarios under which an unmeasured confounder at a prevalence of 40% would fully explain each observed association (eFigure 3 in the Supplement).

## Discussion

Young people initiating benzodiazepines for common sleep conditions had an increased risk of drug overdose during the 6 months after initiation compared with those prescribed alternative pharmacologic treatments. The risk was highest for young people starting benzodiazepine treatment with a recent opioid prescription. Given the availability of alternative pharmacologic and nonpharmacologic treatments for sleep disorders,^5^ our results suggest that using nonbenzodiazepine treatments may reduce drug overdoses in this population.

Concurrent opioid and benzodiazepine use is associated with increased overdose and mortality risks,^27,41,42^ including among adolescents and young adults.^41^ We observed an elevated drug overdose risk for youths with sleep disorders treated with benzodiazepines and recent opioid prescriptions compared with those without opioid prescriptions, as well as for youths prescribed alternative treatments. For young people with insomnia who have not responded to nonpharmacologic interventions, nonbenzodiazepine sleep medication should be strongly considered for those with recent opioid prescriptions. Because of heightened overdose risks among opioid users concomitantly using Z-drugs,^43,44^ cautious prescribing is warranted for current prescription opioid users seeking insomnia treatment.

An association of benzodiazepine treatment with drug overdose was observed for young adults aged 18 to 29 years but was less clear for younger individuals aged 10 to 17 years. The stronger association for young adults may be due to access to and use of substances that, when taken with benzodiazepines, increase drug overdose risk. For example, past-year opioid misuse was higher among individuals aged 18 to 25 years (5.3%) than among individuals aged 12 to 17 years (2.3%).^32^ Physicians may be more cautious in prescribing sleep medications to adolescents given the lack of evidence for sleep medications for this age group.^36^ This approach may result in pharmacologic treatment for insomnia prescribed to adolescents with higher severity of sleep disorders or comorbidities, which could be associated with their observed higher drug overdose incidence. The heterogeneity in treatment practices and drug overdose risk among people aged 10 to 29 years calls for further investigation to explore benzodiazepine treatment and drug overdose risk within narrow age strata.

Although drug overdose risk was elevated for young people initiating benzodiazepines, drug overdose events were also observed among those starting comparator medications. Psychiatric conditions often co-occur with insomnia,^6,45^ which may contribute to overdose risk. Although our analysis focused on overdose risk, other safety concerns should guide prescribing decisions. For example, sleep medications have been associated with suicide attempts,^46^ and Z-drugs carry a boxed warning of serious injuries from complex sleep behaviors.^47^

By aggregating youths initiating hydroxyzine, trazodone, and Z-drugs, we did not estimate 1-to-1 treatment comparisons. There is clinical ambiguity in selecting a prescription medication for insomnia among young people. The comparability of benzodiazepine initiators vs initiators of each medication in our comparator group, based on measured covariates, demonstrated clinical equipoise and provided support for the composite comparator group. The active comparator design reduces potential confounding by indication. Future research should examine risks of sleep disorder treatments separately because little is known concerning overdose risk for these medications in young people, to our knowledge.

Given the frequent concomitant use of benzodiazepines with other substances, it is important to discuss with young people the potential associated harms.^48^ Reviewing patient medications, such as opioids, at prescribing and examining refill needs and whether prescriptions have run out early may help guide initial medication choice and follow-up care. Because other substance use may be unknown to the prescriber, adolescents should be routinely screened for substance use and a history of overdoses before prescribing insomnia treatment.^8^

### Limitations

This study has some limitations. There may be residual confounding by alcohol and drug use, to the extent that this is associated with initial pharmacological treatment. Although the post hoc sensitivity analysis found that a strong unmeasured confounder could explain the results, unmeasured substance use would likely not reach the levels identified because diagnosed substance use disorders and prior poisonings were similar, or less prevalent, among benzodiazepine users than among the comparator group. Our analyses are limited to privately insured individuals; results may differ for patients with Medicaid or uninsured patients, and young adults may transition off their parent’s insurance plan. We measured overdoses treated in a hospital or ED setting. Overdoses that did not result in medical encounters were missed, including fatal overdoses among individuals who did not first present to the ED. The outcome for overdoses involving benzodiazepines is limited to overdoses in which a benzodiazepine was recorded as such; many benzodiazepine overdoses involve multiple substances, and all substances may not be recorded. We examined drug overdose regardless of recorded intent; future investigations considering intent may further guide treatment. The study cohort excluded benzodiazepines, Z-drugs, hydroxyzine, trazodone, and clonidine in the washout period. Other prescribed or over-the-counter medications may also have been used for insomnia. We were unable to measure or account for over-the-counter sleep aids. Benzodiazepines may have been initiated for other indications, and we did not consider whether specific benzodiazepine details, such as dosage or individual benzodiazepines, altered drug overdose risk. Changes in the overdose epidemic may alter risks with benzodiazepine treatment, and our study covers the beginning of the increase in synthetic opioid overdoses.^49^ Finally, our cohort included youths with an insomnia or unspecified sleep disorder diagnosis code; no clinical details were available.

## Conclusions

When treating young people for sleep disorders, it is important to consider the elevated risk of drug overdose with benzodiazepine treatment compared with alternative pharmacologic treatments. Adolescence and young adulthood are critical periods for which rigorous information is needed on the risks of benzodiazepine and other treatments for sleep disorders given the consequences of sleep disorders and the potential for treating insomnia to reduce adverse outcomes.^2,4^ With the lack of large, head-to-head trials of benzodiazepine and alternative sleep medications among young people, continued observational research on the comparative safety of pharmacologic treatments for sleep disorders is necessary to guide treatment decisions. By quantifying the 6-month risk of drug overdose after benzodiazepine treatment initiation for sleep disorders and providing comparative estimates on drug overdose among initiators of other pharmacologic interventions, we hope to inform prescribing decisions and encourage close monitoring for this young patient population.

## Notes

Supplement. **eTable 1.** *ICD-9-CM* and *ICD-10-CM* Codes Used in Outcome Definition **eTable 2.** Young People Initiating BZD or Comparator Treatment for Sleep Disorder: Crude and Propensity Score Weighted Cohorts, Full List of Variables **eTable 3.** Cumulative Incidence and Hazard Ratio of Overdose Involving BZD Within 6 Months of BZD or Comparator Treatment Initiation in Young People With Sleep Disorders **eFigure 1.** Study Flow Diagram **eFigure 2.** Survival Curves of Drug Overdose by Initial Treatment: BZD vs Alternative Sleep Medications **eFigure 3.** Sensitivity Analysis of Residual Confounding (Rule Out Approach)
