# Supplementary material for: Association of Benzodiazepine Treatment for Sleep Disorders With Drug Overdose Risk Among Young People
Source: JAMA Netw Open. 2022 Nov 22;5(11):e2243215. doi: 10.1001/jamanetworkopen.2022.43215 (PMC9682430; doi:10.1001/jamanetworkopen.2022.43215)
Supplement: Supplement. — eTable 1. ICD-9-CM and ICD-10-CM Codes Used in Outcome Definition eTable 2. Young People Initiating BZD or Comparator Treatment for Sleep Disorder: Crude and Propensity Score Weighted Cohorts, Full List of Variables eTable 3. Cumulative Incidence and Hazard Ratio of Overdose Involving BZD Within 6 Months of BZD or Comparator Treatment Initiation in Young People With Sleep Disorders eFigure 1. Study Flow Diagram eFigure 2. Survival Curves of Drug Overdose by Initial Treatment: BZD vs Alternative Sleep Medications eFigure 3. Sensitivity Analysis of Residual Confounding (Rule Out Approach) [file jamanetwopen-e2243215-s001.pdf]

Supplementary Online Content

Bushnell GA, Gerhard T, Keyes K, Hasin D, Cerdá M, Olfson M. Association of benzodiazepine treatment for sleep disorders with drug overdose risk among young people. *JAMA Netw Open.* 2022;5(11):e2243215. doi:10.1001/jamanetworkopen.2022.43215

eTable 1. *ICD-9-CM* and *ICD-10-CM* Codes Used in Outcome Definition
eTable 2. Young People Initiating BZD or Comparator Treatment for Sleep Disorder: Crude and Propensity Score Weighted Cohorts, Full List of Variables
eTable 3. Cumulative Incidence and Hazard Ratio of Overdose Involving BZD Within 6 Months of BZD or Comparator Treatment Initiation in Young People With Sleep Disorders
eFigure 1. Study Flow Diagram
eFigure 2. Survival Curves of Drug Overdose by Initial Treatment: BZD vs Alternative Sleep Medications
eFigure 3. Sensitivity Analysis of Residual Confounding (Rule Out Approach)

This supplementary material has been provided by the authors to give readers additional information about their work.

© 2022 Bushnell GA et al. *JAMA Network Open.*

eTable 1. *ICD-9-CM* and *ICD-10-CM* Codes Used in Outcome Definition

	***ICD-9-CM* codes**	***ICD-10-CM* codes**
Drug overdose	• 960-979.x<br>• E850-E858<br>• E950.0-E950.5<br>• E980.0-E980.5	Codes in the range of T36-T50 that have the 'initial' specification for accidental, intentional, and undetermined poisonings (Excluded codes for 'subsequent encounters' and 'sequala' along with poisonings by assault, adverse effect, and underdosing)
Overdose involving BZD	• 969.4<br>• E853.2	• T42.4X1A<br>• T42.4X2A<br>• T42.4X4A

© 2022 Bushnell GA et al. *JAMA Network Open.*

eTable 2. Young People Initiating BZD or Comparator Treatment for Sleep Disorder: Crude and Propensity Score Weighted Cohorts, Full List of Variables[a]

Patient characteristics	Study cohort, unweighted			IPTW		
	BZD initiators N=23,084 No (%)	Comparator group N=66,706 No (%)	sdiff	BZD initiators N=89,515 Weighted No (%)	Comparator group N=89,814 Weighted No (%)	sdiff
Male	8,640 (37.4)	28,260 (42.4)	0.10	36,674 (41.0)	36,812 (41.0)	0.00
Age at treatment initiation						
10-17y	2,001 (8.7)	9,730 (14.6)	0.19	11,865 (13.3)	11,713 (13.0)	0.01
18-24y	11,424 (49.5)	33,481 (50.2)	0.01	44,712 (49.9)	44,868 (50.0)	0.00
25-29y	9,659 (41.8)	23,495 (35.2)	0.14	32,938 (36.8)	33,233 (37.0)	0.00
Unspecified sleep disorder (vs. insomnia)	2,438 (10.6)	6,255 (9.4)	0.04	8,761 (9.8)	8,690 (9.7)	0.00
**Comorbid psychiatric diagnoses, 1 year**						
ADHD						
Recent, 0-30d	1,275 (5.5)	4,815 (7.2)	0.07	6,156 (6.9)	6,064 (6.8)	0.01
Past (no recent), 31-365d	880 (3.8)	2,626 (3.9)	0.01	3,702 (4.1)	3,526 (3.9)	0.01
None	20,929 (90.7)	59,265 (88.8)	0.06	79,656 (89.0)	80,225 (89.3)	0.01
Major depressive disorder						
Recent, 0-30d	4,045 (17.5)	12,586 (18.9)	0.04	17,011 (19.0)	16,625 (18.5)	0.01
Past (no recent), 31-365d	1,350 (5.8)	4,260 (6.4)	0.02	5,745 (6.4)	5,639 (6.3)	0.01
None	17,689 (76.6)	49,860 (74.7)	0.04	66,759 (74.6)	67,550 (75.2)	0.02
Other depressive disorder	1,311 (5.7)	3,171 (4.8)	0.04	4,733 (5.3)	4,539 (5.1)	0.01
Anxiety disorder, unspecified						
Recent, 0-30d	6,897 (29.9)	10,193 (15.3)	0.36	17,116 (19.1)	17,328 (19.3)	0.00
Past (no recent), 31-365d	1,110 (4.8)	3,778 (5.7)	0.04	5,125 (5.7)	4,885 (5.4)	0.01
None	15,077 (65.3)	52,735 (79.1)	0.31	67,274 (75.2)	67,601 (75.3)	0.00
Generalized anxiety disorder						
Recent, 0-30d	2,660 (11.5)	4,913 (7.4)	0.14	7,670 (8.6)	7,734 (8.6)	0.00
Past (no recent), 31-365d	551 (2.4)	1,872 (2.8)	0.03	2,578 (2.9)	2,419 (2.7)	0.01
None	19,873 (86.1)	59,921 (89.8)	0.12	79,267 (88.6)	79,662 (88.7)	0.01
Panic disorder	1,357 (5.9)	1,361 (2.0)	0.20	2,782 (3.1)	2,828 (3.1)	0.00
Anxiety disorder, other	1,066 (4.6)	2,815 (4.2)	0.02	4,127 (4.6)	3,960 (4.4)	0.01
Adjustment disorder with anxiety or depression	1,599 (6.9)	4,102 (6.1)	0.03	5,808 (6.5)	5,714 (6.4)	0.01
Adjustment disorder, other	528 (2.3)	1,572 (2.4)	0.01	2,143 (2.4)	2,107 (2.3)	0.00
Obsessive compulsive disorder	268 (1.2)	565 (0.8)	0.03	907 (1.0)	863 (1.0)	0.01
Post-traumatic stress disorder	408 (1.8)	1,138 (1.7)	0.01	1,627 (1.8)	1,572 (1.8)	0.01
Autism, pervasive developmental disorder	128 (0.6)	375 (0.6)	0.00	597 (0.7)	515 (0.6)	0.01
Schizophrenia	143 (0.6)	259 (0.4)	0.03	528 (0.6)	444 (0.5)	0.01
Bipolar disorder	749 (3.2)	1,898 (2.8)	0.02	2,845 (3.2)	2,683 (3.0)	0.01
Personality disorder	109 (0.5)	374 (0.6)	0.01	564 (0.6)	485 (0.5)	0.01
Other psychoses	371 (1.6)	826 (1.2)	0.03	1,361 (1.5)	1,239 (1.4)	0.01
Eating disorder	162 (0.7)	531 (0.8)	0.01	686 (0.8)	692 (0.8)	0.00
Other episodic mood disorder	633 (2.7)	2,029 (3.0)	0.02	2,728 (3.0)	2,651 (3.0)	0.01
Conduct/disruptive disorder	214 (0.9)	854 (1.3)	0.03	1,113 (1.2)	1,049 (1.2)	0.01
Acute stress	635 (2.8)	923 (1.4)	0.10	1,613 (1.8)	1,584 (1.8)	0.00
Narcolepsy, cataplexy, hypersomnia	167 (0.7)	443 (0.7)	0.01	639 (0.7)	609 (0.7)	0.00
Other sleep disorder	306 (1.3)	846 (1.3)	0.01	1,307 (1.5)	1,165 (1.3)	0.01
Suicidal ideation diagnosis	394 (1.7)	1,894 (2.8)	0.08	2,340 (2.6)	2,223 (2.5)	0.01

© 2022 Bushnell GA et al. *JAMA Network Open.*

Self-harm diagnosis	71 (0.3)	478 (0.7)	0.06	472 (0.5)	499 (0.6)	0.00
Tobacco use disorder						
Recent, 0-30d	463 (2.0)	1,429 (2.1)	0.01	1,915 (2.1)	1,890 (2.1)	0.00
Past (no recent), 31-365d	890 (3.9)	2,320 (3.5)	0.02	3,278 (3.7)	3,237 (3.6)	0.00
None	21,731 (94.1)	62,957 (94.4)	0.01	84,322 (94.2)	84,688 (94.3)	0.00
Alcohol use disorder	462 (2.0)	1,620 (2.4)	0.03	2,121 (2.4)	2,079 (2.3)	0.00
Cannabis use disorder	404 (1.8)	1,631 (2.4)	0.05	2,182 (2.4)	2,040 (2.3)	0.01
Opioid use disorder	253 (1.1)	943 (1.4)	0.03	1,263 (1.4)	1,198 (1.3)	0.01
Stimulant use disorder	98 (0.4)	404 (0.6)	0.03	499 (0.6)	492 (0.5)	0.00
Sedative use disorder	58 (0.3)	230 (0.3)	0.02	276 (0.3)	276 (0.3)	0.00
Other substance use disorder	228 (1.0)	524 (0.8)	0.02	775 (0.9)	759 (0.8)	0.00
**Medications, 1 year**						
SSRI						
Recent, 0-3mo	8,629 (37.4)	18,547 (27.8)	0.21	27,352 (30.6)	27,444 (30.6)	0.00
Past (no recent), 3-12mo	927 (4.0)	2,856 (4.3)	0.01	3,952 (4.4)	3,789 (4.2)	0.01
None	13,528 (58.6)	45,303 (67.9)	0.19	58,210 (65.0)	58,581 (65.2)	0.00
Opioid (prescription)						
Recent, 0-3mo	3,504 (15.2)	8,867 (13.3)	0.05	12,817 (14.3)	12,469 (13.9)	0.01
Past (no recent), 3-12mo	4,653 (20.2)	13,038 (19.5)	0.02	17,603 (19.7)	17,689 (19.7)	0.00
None	14,927 (64.7)	44,801 (67.2)	0.05	59,095 (66.0)	59,657 (66.4)	0.01
Skeletal muscle relaxant						
Recent, 0-3mo	1,054 (4.6)	2,848 (4.3)	0.01	3,944 (4.4)	3,919 (4.4)	0.00
Past (no recent), 3-12mo	1,271 (5.5)	3,766 (5.6)	0.01	5,183 (5.8)	5,050 (5.6)	0.01
None	20,759 (89.9)	60,092 (90.1)	0.01	80,388 (89.8)	80,845 (90.0)	0.01
SNRI	1,183 (5.1)	3,095 (4.6)	0.02	4,581 (5.1)	4,346 (4.8)	0.01
Non-SSRI/SNRI antidepressant	2,450 (10.6)	6,494 (9.7)	0.03	9,225 (10.3)	9,034 (10.1)	0.01
Stimulant	2,198 (9.5)	7,131 (10.7)	0.04	9,366 (10.5)	9,353 (10.4)	0.00
Antipsychotic	1,164 (5.0)	2,469 (3.7)	0.07	3,970 (4.4)	3,744 (4.2)	0.01
Hypnotic, sedative	702 (3.0)	1,923 (2.9)	0.01	2,896 (3.2)	2,661 (3.0)	0.02
Mood stabilizer, lithium	650 (2.8)	1,650 (2.5)	0.02	2,528 (2.8)	2,338 (2.6)	0.01
Atomoxetine	92 (0.4)	449 (0.7)	0.04	510 (0.6)	527 (0.6)	0.00
Guanfacine	79 (0.3)	393 (0.6)	0.04	476 (0.5)	458 (0.5)	0.00
NSAIDs	4,976 (21.6)	14,195 (21.3)	0.01	19,204 (21.5)	19,213 (21.4)	0.00
Asthma medication	4,760 (20.6)	14,075 (21.1)	0.01	18,895 (21.1)	18,897 (21.0)	0.00
Antihistamine	1,970 (8.5)	5,558 (8.3)	0.01	7,796 (8.7)	7,562 (8.4)	0.01
Beta-blockers	773 (3.3)	1,953 (2.9)	0.02	2,938 (3.3)	2,764 (3.1)	0.01
Cardiovascular medications	732 (3.2)	2,240 (3.4)	0.01	3,092 (3.5)	2,996 (3.3)	0.01
Other anticonvulsants	543 (2.4)	1,497 (2.2)	0.01	2,185 (2.4)	2,072 (2.3)	0.01
Gabapentin, pregabalin	460 (2.0)	1,431 (2.1)	0.01	2,096 (2.3)	1,912 (2.1)	0.01
Antidiabetics	387 (1.7)	1,242 (1.9)	0.01	1,634 (1.8)	1,628 (1.8)	0.00
**Healthcare utilization, 1 year**						
Inpatient psychiatric admission	677 (2.9)	2,953 (4.4)	0.08	3,766 (4.2)	3,565 (4.0)	0.01
Inpatient non-psychiatric admission	1,222 (5.3)	2,809 (4.2)	0.05	4,094 (4.6)	4,054 (4.5)	0.00
Emergency department visit						
Recent, 0-3mo	3,392 (14.7)	8,499 (12.7)	0.06	12,288 (13.7)	11,939 (13.3)	0.01
Past (no recent), 3-12mo	3,417 (14.8)	10,559 (15.8)	0.03	14,024 (15.7)	14,011 (15.6)	0.00
None	16,275 (70.5)	47,648 (71.4)	0.02	63,203 (70.6)	63,865 (71.1)	0.01
Psychotherapy claim						
Recent, 0-1mo	1,941 (8.4)	5,786 (8.7)	0.01	8,337 (9.3)	7,816 (8.7)	0.02
Past (no recent), 1-12mo	1,382 (6.0)	4,702 (7.0)	0.04	6,296 (7.0)	6,081 (6.8)	0.01
None	19,761 (85.6)	56,218 (84.3)	0.04	74,881 (83.7)	75,918 (84.5)	0.02
Outpatient visit count						
0-4 visits	7,487 (32.4)	22,097 (33.1)	0.02	28,518 (31.9)	29,457 (32.8)	0.02
5-10 visits	8,270 (35.8)	23,436 (35.1)	0.01	31,565 (35.3)	31,693 (35.3)	0.00
11+ visits	7,327 (31.7)	21,173 (31.7)	0.00	29,431 (32.9)	28,664 (31.9)	0.02
Preventative/well visit	10,347 (44.8)	29,372 (44.0)	0.02	39,713 (44.4)	39,777 (44.3)	0.00

© 2022 Bushnell GA et al. *JAMA Network Open.*

Recent provider contact						
Psychiatry	1,535 (6.6)	4,843 (7.3)	0.02	6,742 (7.5)	6,408 (7.1)	0.02
Other mental health provider	2,041 (8.8)	6,029 (9.0)	0.01	8,680 (9.7)	8,124 (9.0)	0.02
Neurology	726 (3.1)	1,497 (2.2)	0.06	2,447 (2.7)	2,268 (2.5)	0.01
**Other diagnoses, 1 year**						
Musculoskeletal pain	5,312 (23.0)	16,206 (24.3)	0.03	21,829 (24.4)	21,566 (24.0)	0.01
Fatigue, malaise	5,228 (22.6)	12,528 (18.8)	0.10	17,871 (20.0)	17,855 (19.9)	0.00
Migraine, headache	4,112 (17.8)	11,590 (17.4)	0.01	16,017 (17.9)	15,781 (17.6)	0.01
Joint disorders and dislocations	3,457 (15.0)	10,857 (16.3)	0.04	14,547 (16.3)	14,346 (16.0)	0.01
Allergic rhinitis	2,814 (12.2)	8,337 (12.5)	0.01	11,115 (12.4)	11,185 (12.5)	0.00
Low back pain	2,243 (9.7)	6,486 (9.7)	0.00	8,837 (9.9)	8,741 (9.7)	0.01
Nonspecific chest pain	1,890 (8.2)	4,373 (6.6)	0.06	6,484 (7.2)	6,338 (7.1)	0.01
Asthma	1,746 (7.6)	5,331 (8.0)	0.02	7,099 (7.9)	7,092 (7.9)	0.00
Syncope, dizziness	1,543 (6.7)	3,753 (5.6)	0.04	5,404 (6.0)	5,357 (6.0)	0.00
Obesity	1,368 (5.9)	4,431 (6.6)	0.03	5,758 (6.4)	5,789 (6.4)	0.00
Palpitations	1,185 (5.1)	1,865 (2.8)	0.12	3,084 (3.4)	3,125 (3.5)	0.00
Pregnancy	1,091 (4.7)	2,326 (3.5)	0.06	3,383 (3.8)	3,430 (3.8)	0.00
Anemia	1,059 (4.6)	2,399 (3.6)	0.05	3,575 (4.0)	3,509 (3.9)	0.00
Myalgia, fibromyalgia, myositis	994 (4.3)	2,687 (4.0)	0.01	3,824 (4.3)	3,716 (4.1)	0.01
Muscle spasm	986 (4.3)	2,530 (3.8)	0.02	3,577 (4.0)	3,521 (3.9)	0.00
Cancer, malignancy	869 (3.8)	2,102 (3.2)	0.03	3,009 (3.4)	2,963 (3.3)	0.00
Nervous system pain, pain syndromes	630 (2.7)	1,855 (2.8)	0.00	2,525 (2.8)	2,492 (2.8)	0.00
IBS, Crohn's disease	630 (2.7)	1,608 (2.4)	0.02	2,396 (2.7)	2,279 (2.5)	0.01
Hypertension	602 (2.6)	1,392 (2.1)	0.03	2,039 (2.3)	2,007 (2.2)	0.00
Overweight	506 (2.2)	1,777 (2.7)	0.03	2,130 (2.4)	2,274 (2.5)	0.01
Cardiac dysrhythmias	488 (2.1)	1,069 (1.6)	0.04	1,626 (1.8)	1,588 (1.8)	0.00
Diabetes	446 (1.9)	1,106 (1.7)	0.02	1,525 (1.7)	1,553 (1.7)	0.00
Sexually transmitted infection	445 (1.9)	1,214 (1.8)	0.01	1,646 (1.8)	1,659 (1.8)	0.00
Premenstrual dysphoric disorder	146 (0.6)	292 (0.4)	0.03	439 (0.5)	441 (0.5)	0.00
Low blood pressure	118 (0.5)	290 (0.4)	0.01	419 (0.5)	419 (0.5)	0.00
Pain, unspecified	304 (1.3)	806 (1.2)	0.01	1,168 (1.3)	1,114 (1.2)	0.01
Scoliosis	291 (1.3)	892 (1.3)	0.01	1,213 (1.4)	1,191 (1.3)	0.00
Fracture	698 (3.0)	2,219 (3.3)	0.02	3,041 (3.4)	2,934 (3.3)	0.01
Injury to body (excluding fractures)	4,791 (20.8)	14,819 (22.2)	0.04	19,836 (22.2)	19,639 (21.9)	0.01
Injury, other	2,142 (9.3)	5,894 (8.8)	0.02	8,147 (9.1)	8,068 (9.0)	0.00
Poisoning, adverse effect						
Recent, 0-30d	203 (0.9)	744 (1.1)	0.02	916 (1.0)	925 (1.0)	0.00
Past (no recent), 31-365d	182 (0.8)	564 (0.8)	0.01	792 (0.9)	761 (0.8)	0.00
None	22,699 (98.3)	65,398 (98.0)	0.02	87,806 (98.1)	88,129 (98.1)	0.00
**Other variables**						
Geographical division						
East North Central	3,456 (15.0)	10,579 (15.9)	0.03	14,269 (15.9)	14,034 (15.6)	0.01
East South Central	1,435 (6.2)	5,170 (7.8)	0.06	6,526 (7.3)	6,612 (7.4)	0.00
Mid-Atlantic	2,750 (11.9)	5,843 (8.8)	0.10	8,478 (9.5)	8,597 (9.6)	0.00
Mountain	1,872 (8.1)	5,684 (8.5)	0.02	7,379 (8.2)	7,541 (8.4)	0.01
New England	932 (4.0)	2,001 (3.0)	0.06	2,932 (3.3)	2,945 (3.3)	0.00
Pacific	2,784 (12.1)	8,317 (12.5)	0.01	11,077 (12.4)	11,104 (12.4)	0.00
South Atlantic	4,506 (19.5)	11,684 (17.5)	0.05	16,142 (18.0)	16,234 (18.1)	0.00
West North Central	970 (4.2)	4,059 (6.1)	0.09	5,028 (5.6)	5,003 (5.6)	0.00
West South Central	3,680 (15.9)	10,869 (16.3)	0.01	14,408 (16.1)	14,540 (16.2)	0.00
Unknown	699 (3.0)	2,500 (3.7)	0.04	3,274 (3.7)	3,205 (3.6)	0.01
First new use period in dataset	19,211 (83.2)	57,103 (85.6)	0.07	75,890 (84.8)	76,263 (84.9)	0.00
Provider/facility diagnosing sleep disorder						
Family practitioner	10,882 (47.1)	32,164 (48.2)	0.02	42,640 (47.6)	43,094 (48.0)	0.01
MD, not elsewhere classified	5,018 (21.7)	11,489 (17.2)	0.11	16,430 (18.4)	16,490 (18.4)	0.00

© 2022 Bushnell GA et al. *JAMA Network Open.*

Nurse practitioner, Physician's Assistant	754 (3.3)	3,389 (5.1)	0.09	4,102 (4.6)	4,120 (4.6)	0.00
Pediatrician	576 (2.5)	2,687 (4.0)	0.09	3,288 (3.7)	3,244 (3.6)	0.00
Acute care hospital	592 (2.6)	1,588 (2.4)	0.01	2,101 (2.3)	2,173 (2.4)	0.01
Other	3,420 (14.8)	10,030 (15.0)	0.01	13,801 (15.4)	13,503 (15.0)	0.01
Unknown or multiple providers	1,842 (8.0)	5,359 (8.0)	0.00	7,153 (8.0)	7,191 (8.0)	0.00

ADHD: Attention-deficit/hyperactivity disorder; BZD: benzodiazepine; d: day; ED: Emergency department; IBS: Irritable bowel syndrome; IPTW: Inverse probability of treatment weighting; MD: medical doctor; mo: months; NSAID: Nonsteroidal anti-inflammatory drug; SDIFF: Standardized difference; SSRI: Selective serotonin reuptake inhibitor; SNRI: Serotonin–norepinephrine reuptake inhibitor; y: year

2009-2018 MarketScan commercial claims database

[a]All variables were included in the propensity score estimation

© 2022 Bushnell GA et al. *JAMA Network Open.*

eTable 3. Cumulative Incidence and Hazard Ratio of Overdose Involving BZD Within 6 Months of BZD or Comparator Treatment Initiation in Young People With Sleep Disorders[a]

	Total No. (unweighted)	BZD overdose events[b]	Cumulative incidence		Hazard ratio (95% CI)
			6mo	Difference at 6mo	
**Intention-to-treat analysis**					
Crude					
BZD treatment	23,084	70	0.33%	0.25%	4.40 (3.03 – 6.38)
Comparator treatment	66,706	46	0.08%	REF	REF
Adjusted					
BZD treatment	23,084	275.4	0.33%	0.26%	4.65 (3.13 – 6.91)
Comparator treatment	66,553	59.4	0.07%	REF	REF
**As-treated analysis**					
Crude					
BZD treatment	23,084	54	0.56%	0.49%	7.14 (4.41-11.56)
Comparator treatment	66,706	24	0.06%	REF	REF
Adjusted					
BZD treatment	23,084	217.9	0.64%	0.58%	7.67 (4.61-12.76)
Comparator treatment	66,553	32.0	0.06%	REF	REF

BZD: benzodiazepine; CI: confidence interval; mo: months; REF: reference
[a]Young people aged 10-29 years with an insomnia or unspecific sleep disorder diagnosis
[b]Weighted outcome counts displayed for adjusted analyses

© 2022 Bushnell GA et al. *JAMA Network Open.*

eFigure 1. Study Flow Diagram

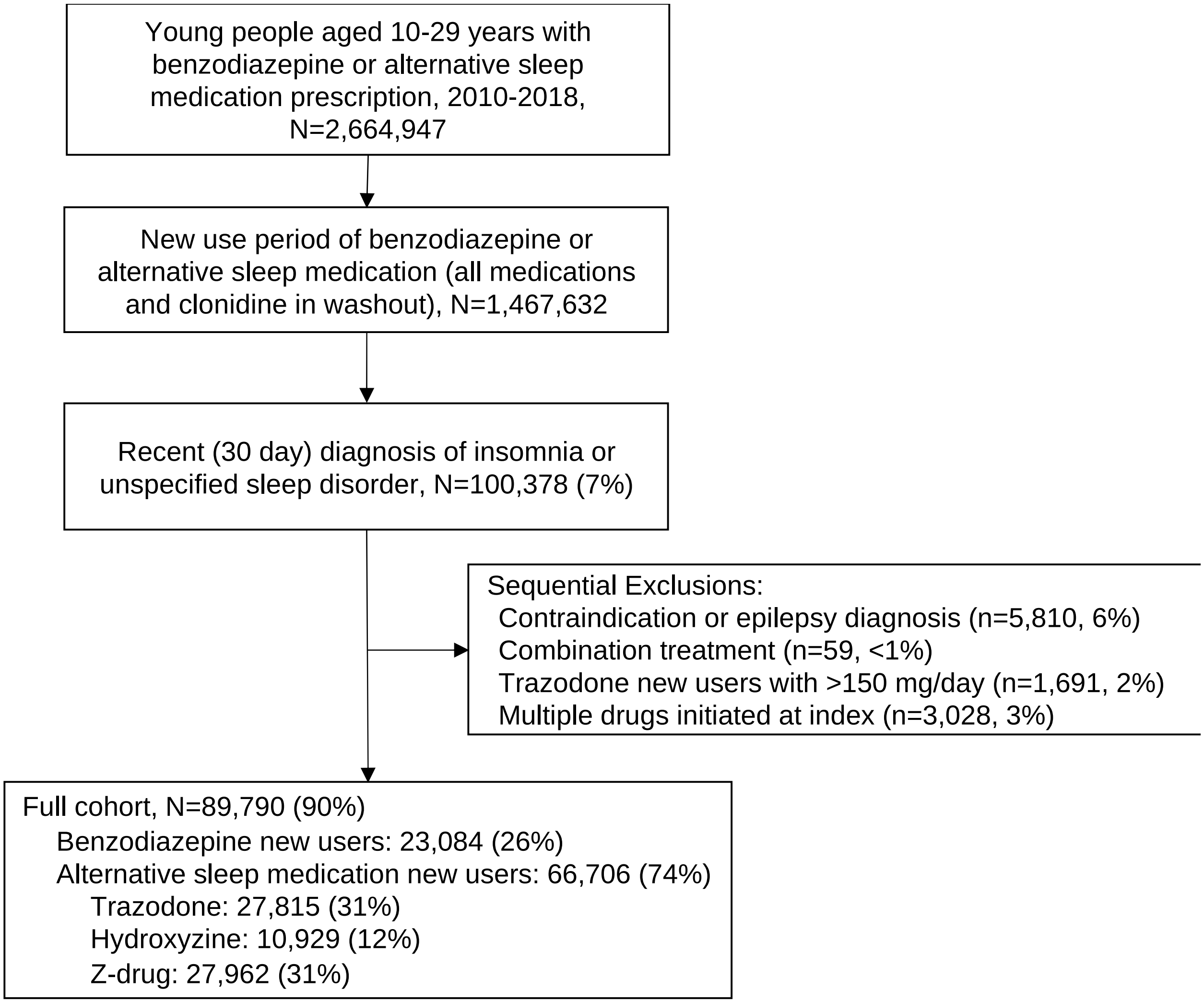

© 2022 Bushnell GA et al. *JAMA Network Open.*

eFigure 2. Survival Curves of Drug Overdose by Initial Treatment: BZD vs Alternative Sleep Medications
A) intention-to-treat analysis and B) as-treated analysis censoring at treatment discontinuation

A) Intention-to-treat

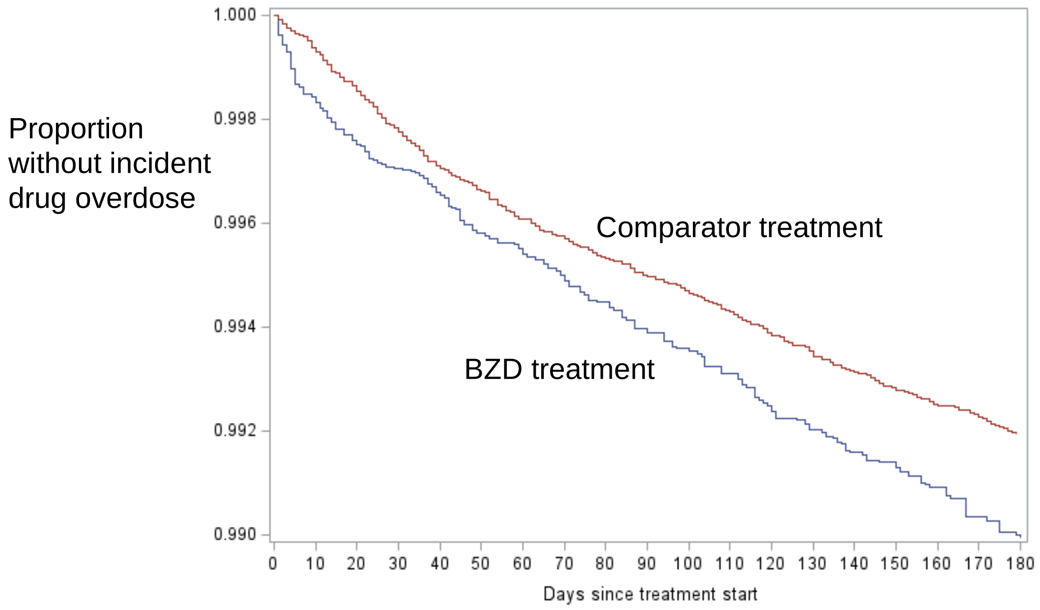

B) As-treated

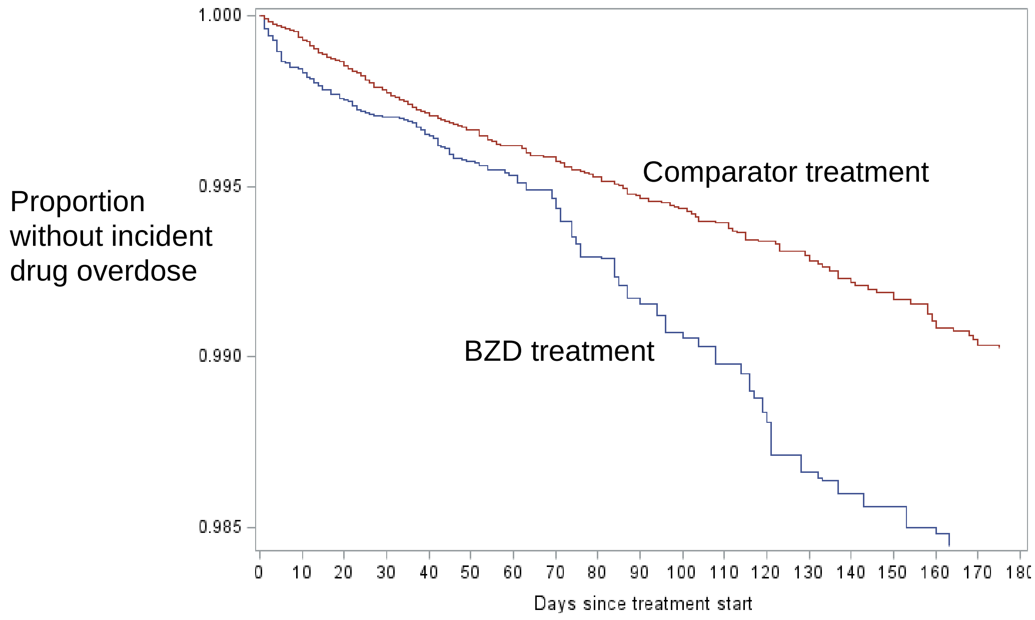

© 2022 Bushnell GA et al. *JAMA Network Open.*

eFigure 3. Sensitivity Analysis of Residual Confounding (Rule Out Approach): Example for RR=1.44 and RR=1.25 assuming unmeasured confounder prevalence at 40%[a,b]

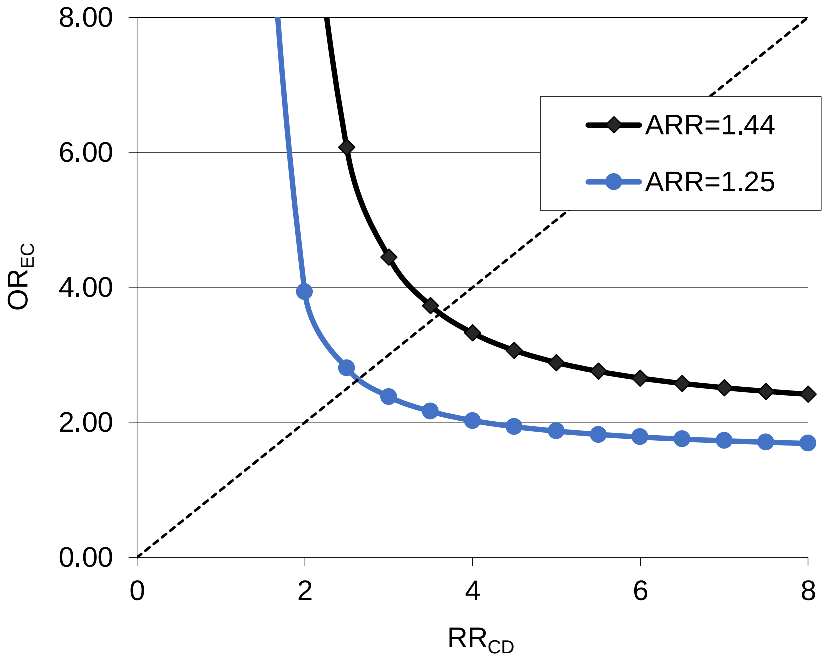

ARR: Observed relative risk; OR_{EC}: Odds ratio between treatment status (benzodiazepine vs. comparator sleep medication) and unmeasured confounder prevalence; RR_{CD}: Association between unmeasured confounder and the study outcome (drug overdose within 6 months).

[a]ARR=1.44 corresponds to the adjusted effect estimate from the primary as-treated analysis and ARR=1.25 corresponds to the adjusted effect estimate from the primary intention-to-treat analysis
[b]The area to the upper right of each curved line represents the combinations of OR_{EC} and RR_{CD} in which confounding by an unmeasured factor would be strong enough to move the point estimate to null (RR=1.00) or below null. The area to the lower-left of each curved line representations the combinations of OR_{EC} and RR_{CD} in which the point estimate would remain above null (RR>1.00).
[c]Figure adapted from Schneeweiss S. Sensitivity analysis and external adjustment for unmeasured confounders in epidemiologic database studies of therapeutics. *Pharmacoepidemiol Drug Safety*, 2006;15:291-303.

© 2022 Bushnell GA et al. *JAMA Network Open.*
